# Emergency Chest Pain Center: A Novel Approach to Reduce Door to Balloon Time

**DOI:** 10.1016/j.jacadv.2025.101774

**Published:** 2025-05-13

**Authors:** Marwa A. Sabe, Frank J. Kaeberlein, Sharif A. Sabe, Allyson Kelly, Tracy Summerfield, Ahmed A. Sabe

**Affiliations:** a Beth Israel Deaconess Medical Center, Harvard Medical School, Boston, Massachusetts, USA; b Emergency Services Institute, Cleveland Clinic Mercy Hospital, Cleveland Clinic Foundation, Cleveland, Ohio, USA; c Mercy Cardiovascular Institute, Cleveland Clinic Mercy Hospital, Cleveland Clinic Foundation, Cleveland, Ohio, USA

**Keywords:** catheterization lab, door to balloon time, emergency room, mortality

## Abstract

**Background:**

Percutaneous coronary intervention is the preferred treatment for acute ST-segment elevation myocardial infarction (STEMI), and shorter door-to-balloon time (D2B) is associated with lower mortality. We implemented a catheterization laboratory within the emergency department (ED) as a novel strategy to reduce D2B.

**Objectives:**

The purpose of this paper was to compare D2B and mortality in STEMI patients presenting to ED vs standard catheterization labs at a community hospital.

**Methods:**

We prospectively reviewed consecutive patients presenting with STEMI to our institution between 1998 and 2011 and treated with primary percutaneous coronary intervention. The primary endpoints were D2B and time to death. A multivariable linear regression model was used to assess the relationship between catheterization lab location and D2B. The relationship between D2B and mortality was examined using a Cox proportional hazards model.

**Results:**

We included 1,053 STEMI patients (553 in ED vs 500 in standard catheterization labs). Both groups had similar age, sex, race, diabetes, left main disease, and Killip class on presentation. Standard catheterization lab patients were more likely to have left ventricular ejection fraction <40% (11% vs 6.5%). D2B was shorter in ED vs standard cath lab patients (54 vs 83 minutes, *P* < 0.001). ED catheterization lab patients were more likely to have <30-minute D2B (17% vs <1%, *P* < 0.001). After covariate adjustment, ED catheterization lab patients had lower 30-day (adjusted hazard ratio [adj HR]: 0.54, 95% confidence interval [CI] 0.29-0.99), 1-year (adj HR: 0.58, 95% CI: 0.37-0.91), and 10-year mortality (adj HR: 0.39, 95% CI: 0.29-0.53) than standard catheterization lab patients.

**Conclusions:**

Implementation of an ED catheterization lab is a feasible strategy which may reduce D2B and STEMI mortality.

Primary percutaneous coronary intervention (PCI) is the treatment of choice for acute ST-segment elevation myocardial infarction (STEMI). In this setting, decreased door-to-balloon (D2B) time is associated with improved patient outcomes. The recommended D2B time is <90 minutes, and every 30-minute increase in D2B time has been shown to increase 1-year mortality by 7.5%.^1^ Park et al^2^ found that every reduction in D2B time by 30 minutes was associated with continuous improvement in 1-year mortality. Furthermore, D2B ≤30 minutes may be associated with improved in-hospital mortality compared to D2B >30 minutes, even if less than the recommended 90-minute threshold.^3^

In an effort to keep D2B <90 minutes, there have been worldwide attempts to reduce D2B time. These include out-of-hospital ECG transmission,^4^^,^^5^ obtaining ECG within 10 minutes of presentation, central paging systems for cath lab activation,^6^ emergency department (ED) triage of patients with early activation of catheterization labs by the ED physicians,^7^ and immediate transfer by an in-house transfer team.^8^ Khot et al^8^ found that among patients presenting with STEMI, ED physician-directed activation of the catheterization lab and ED-mediated transfer to an immediately available catheterization lab resulted in shorter D2B times. Importantly, these strategies were associated with a reduced mean infarct size, length of stay, and total hospital costs per admission.^8^ Therefore, hospital implementation of strategies targeted to reduce D2B times are necessary to improve outcomes in patients with STEMI.

In addition to some of the aforementioned techniques to decrease D2B time, we implemented a catheterization laboratory within the ED at our institution as a novel strategy to further reduce D2B time and improve access to immediate revascularization. In this study, we compare D2B time and mortality in patients presenting with STEMI to the ED-based vs standard catheterization labs at a community hospital. To our knowledge, this is the first reported study of an emergency chest pain center which includes a catheterization lab in the ED.

## Methods

### Study population

We included 1,053 consecutive patients with STEMI who underwent primary angioplasty at our institution between April 1998 and December 2011. Patients were non-randomized to either the ED or standard catheterization laboratory (cath lab). The decision to send the patient to either cath lab was based on physician preference and catheterization laboratory availability. Standard STEMI protocol was utilized in both labs. Patients who refused cardiac catheterization or had a contraindication to cardiac catheterization were excluded from the study. This study was approved by the Cleveland Clinic Mercy Hospital Institutional Review Board (IRB#2019006).

### Variables and outcomes of interest

The exposure of interest was the type of cath lab used (ED vs standard). The primary endpoints were D2B time and time to death (up to 30 days, 1 year, and 10 years). Patient demographics, medications, and past medical history were collected from the electronic medical record. Left ventricular ejection fraction (LVEF) and mitral regurgitation severity were obtained from a 2-dimensional echocardiogram or left ventriculogram that was obtained during the STEMI hospitalization. D2B time (in minutes) was collected at the time of catheterization and calculated as the time from entry into our institution to reperfusion. Mortality data were obtained from the social security death index and the hospital medical record.

### Statistical analysis

Continuous variables were examined for normality and reported as either mean ± SD or median (IQR). Categorical variables were expressed as a number and percent. A Student's *t*-test (for normal variables) and a Wilcoxon rank-sum test (for non-normal variables) were used to compare continuous variables. A Fisher's exact test was used to compare categorical variables.

A multivariable linear regression model was used to assess the relationship between cath lab location and door-to-balloon time in minutes. We included variables that could be confounders of this relationship as well as other predictors of our outcomes. We entered into the model any variable with an association with the outcomes on univariable analysis with a *P* value <0.20 in addition to those which were clinically relevant.

The univariate relationship between cath lab location and the primary outcome time to death (up to 30 days, 1 year, 10 years) was examined using a Kaplan-Meier curve. A Cox proportional hazards model was used to adjust for univariate predictors of mortality. Age, sex, and race were included in the model, as were any predictors with a *P* value < 0.2 on univariate analysis and those that were clinically relevant. Specifically, we included clinical variables that could have an impact on mortality in patients presenting with STEMI, including history of diabetes, hypertension, congestive heart failure, severe mitral regurgitation, and prior coronary artery bypass grafting (CABG). Additional clinical variables at the time of presentation that could impact mortality were included in the model such as cardiogenic shock and the use of intra-aortic balloon pump.

We also examined the relationship between D2B (divided into categories) and mortality using a Cox proportional hazards model and included relevant covariates as noted earlier.

## Results

A total of 1,053 patients who presented with STEMI were included in this analysis; 553 of which were treated in the ED cath lab, and 500 went to the standard cath lab. There was no significant difference in the age (59.7 vs 58.9 years, *P* = 0.29), sex (70% vs 73% male, *P* = 0.31), or race (95% vs 94% white, *P* = 0.31) between the 2 groups (see Table 1). Approximately 20% of all patients had diabetes mellitus (DM), and there were no significant differences in the rate of diabetes between the 2 groups (*P* = 0.4). There was no significant difference between a history of CABG (*P* = 0.8) or left main coronary artery disease on presentation (*P* = 0.7). Patients who presented to the standard cath lab were more likely to have an LVEF <40% (11% vs 6.5%, *P* = 0.005), but there was no difference in Killip class at presentation (*P* = 0.16). Unadjusted outcomes comparing ED and standard cath lab groups are reported in Table 2.

**Table 1:** Baseline Characteristics of ED vs Standard Cath Lab

	ED Cath Lab (n = 553)	Standard Cath Lab (n = 500)	*P* Value
Age, y	59.7 ± 12.9	58.9 ± 12.2	0.29
Sex			0.31
Male	386 (70%)	364 (73%)	
Female	167 (30%)	137 (27%)	
Race			
White	523 (95%)	470 (94%)	
Black	25 (5%)	28 (6%)	
Other	5 (<1%)	2 (<1%)	
History of DM	109 (20%)	88 (18%)	0.37
History of HTN	253 (46%)	214 (43%)	0.35
LVEF <40%	36 (6.5%)	57 (11%)	0.005
Killip Class 3-4	34 (6.1%)	42 (8.4%)	0.16
History of CABG	40 (7.3%)	39 (7.8%)	0.75
Culprit vessel^a^			
LM	7 (1.3%)	5 (1.0%)	
LAD	191 (35%)	175 (35%)	
LCx	86 (16%)	59 (12%)	
RCA	289 (52%)	258 (51.6%)	
SVG	10 (1.8%)	13 (2.6%)	
Ramus	5 (0.9%)	7 (1.4%)	

CABG = coronary artery bypass grafting; DM = diabetes mellitus; ED = emergency department; HTN = hypertension; LAD = left anterior descending coronary artery; LCx = left circumflex coronary artery; LM = left main coronary artery; LVEF = left ventricular ejection fraction; RCA = right coronary artery; SVG = saphenous vein graft; Ramus = ramus intermedius coronary artery.

a Some patients presented with multiple culprit coronary vessels.

**Table 2:** Unadjusted Outcomes Comparing ED vs Standard Cath Lab

	ED Cath Lab (n = 553)	Standard Cath Lab (n = 500)	Unadjusted *P* Value
D2B			<0.001
<30 min	96 (17%)	4 (0.8%)	
30-60 min	232 (42%)	92 (18%)	
>60 min	225 (41%)	404 (81%)	
IABP	137 (25%)	163 (33%)	0.005
LOS post-intervention	4.4 (6.4)	4.4 (4.4)	0.997

cath lab = catheterization laboratory; D2B = door to balloon time; ED = emergency department; IABP = intra-aortic balloon pump placement; LOS = length of stay.

**Figure 1:**
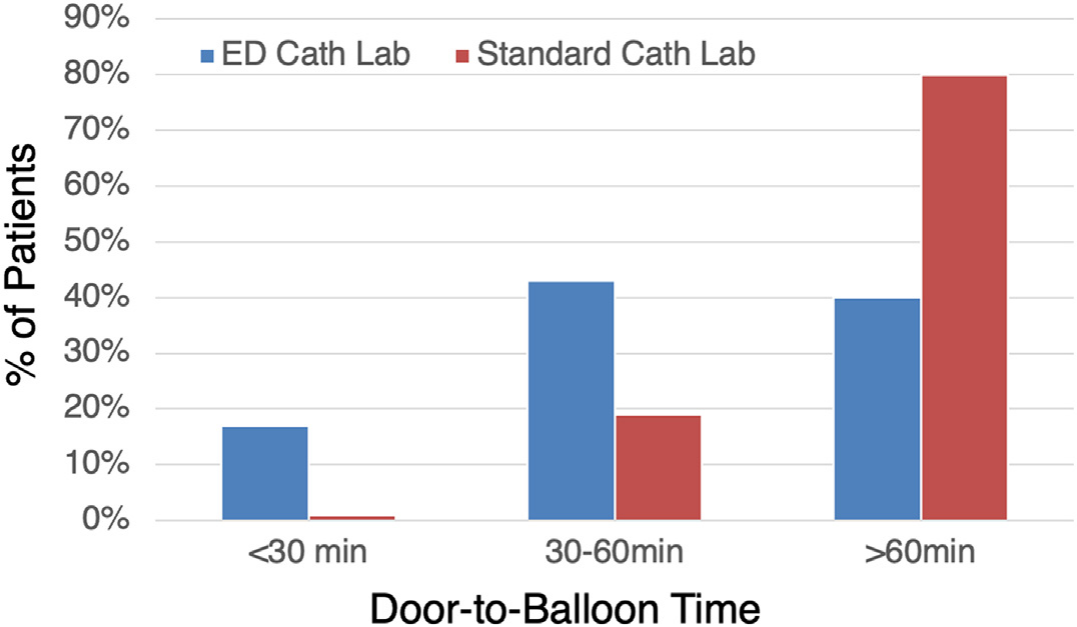
Door-to-Balloon Times in ED and Standard Catheterization Labs Patients presenting with ST-segment elevation myocardial infarction undergoing primary percutaneous intervention had lower door-to-balloon times when treated in the emergency department catheterization lab (ED cath lab, blue bars), compared to standard catheterization lab (standard cath lab, red bars).

After adjusting for covariates, the patients who were treated in the ED cath lab had a significantly lower D2B time than patients treated in the standard cath lab (median 54 vs 83 minutes, *P* < 0.001). When D2B time was split into categories of <30 minutes, 30 to 60 minutes, and ≥60 minutes, patients in the ED cath lab were more likely to have a <30-minute D2B time (17% vs <1%, *P* < 0.001) (Figure 1). Twenty-eight patients had D2B ≤15 minutes, and all these patients were treated in the ED cath lab. The lowest D2B in this study was 6 minutes, and this patient was treated in the ED cath lab.

**Figure 2:**
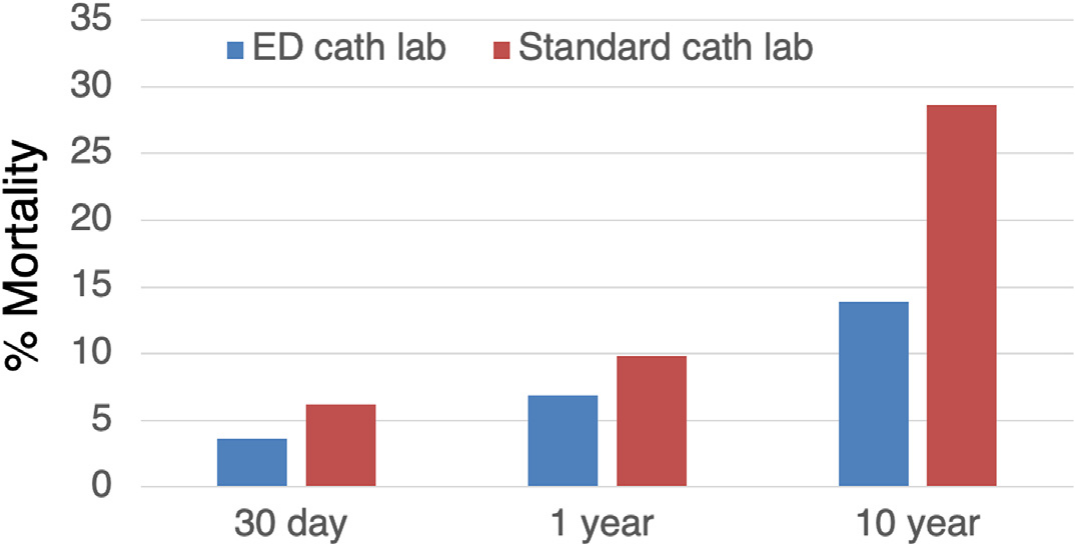
Thirty-Day, 1-Year, and 10-Year Mortality in ED and Standard Catheterization Labs Percent mortality demonstrated in patients treated in emergency department catheterization lab (ED cath lab, blue bars), compared to standard catheterization lab (standard cath lab, red bars).

**Figure 3:**
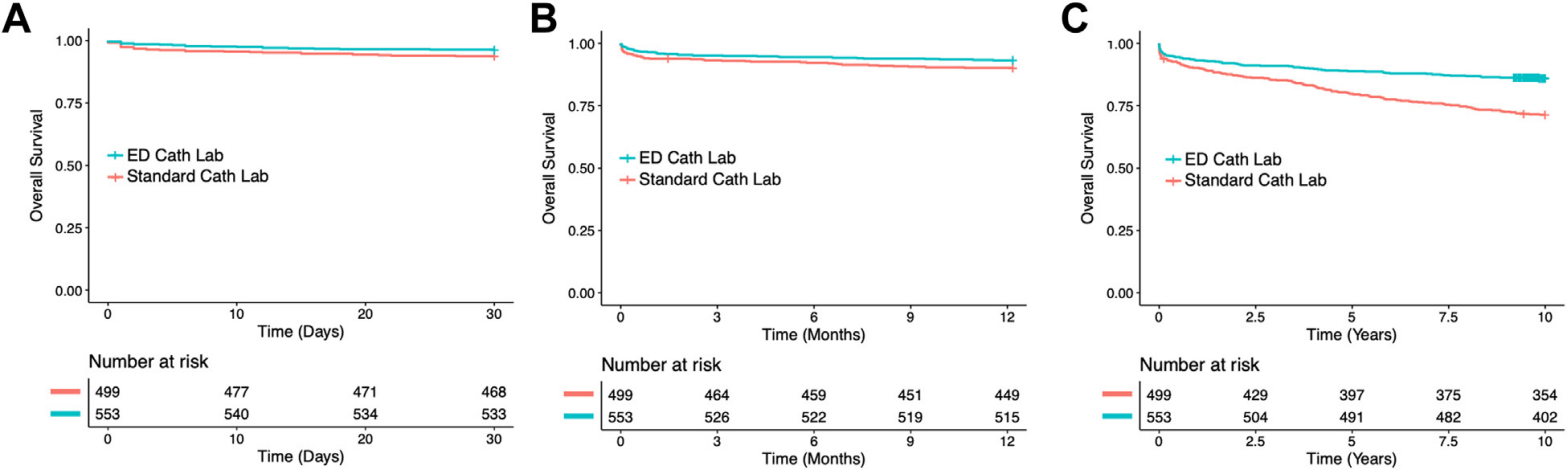
Cox Regression Assessing Short- and Long-Term Mortality in ED vs Standard Catheterization Lab Cox proportional hazards regression plot shown for (A) 30-day mortality, (B) 1-year mortality, and (C) 10-year mortality of patients presenting with ST-segment elevation myocardial infarction undergoing percutaneous intervention in the emergency department catheterization lab (ED cath lab, blue) compared to standard catheterization lab (standard cath lab, red).

After adjusting for age, sex, race, history of DM, history of heart failure, history of CABG, history of hypertension, cardiogenic shock on presentation, the use of intraaortic balloon pump, severe mitral regurgitation, and LVEF <40%, patients who presented to the ED cath lab had a significantly lower 30-day mortality (adjusted hazard ratio [adj HR]: 0.54, 95% CI: 0.29-0.99, *P* = 0.047) and 1-year mortality (adj HR: 0.58, 95% CI: 0.37-0.91, *P* < 0.018) than patients treated in the standard cath lab. Patients who were treated in the ED cath lab also had significantly lower long-term (up to 10 years) mortality than patients treated in the standard cath lab (adj HR: 0.39, 95% CI: 0.29-0.53, *P* < 0.0001) (Table 3, Figures 2 and 3, Figure 2, Figure 3). Other significant predictors of mortality included older age, a diagnosis of DM, LVEF <40%, and cardiogenic shock on presentation (Table 4).

**Table 3:** Adjusted Hazard Ratios Comparing Mortality in ED vs Standard Cath Lab

Survival Follow-Up Time	Adjusted HR for ED Cath Lab vs Standard Cath Lab	*P* Value
30 d	0.54	0.047
1 y	0.58	0.018
10 y	0.39	<0.0001

cath lab = catheterization laboratory; ED = emergency department; HR = hazard ratio.

**Central Illustration:**
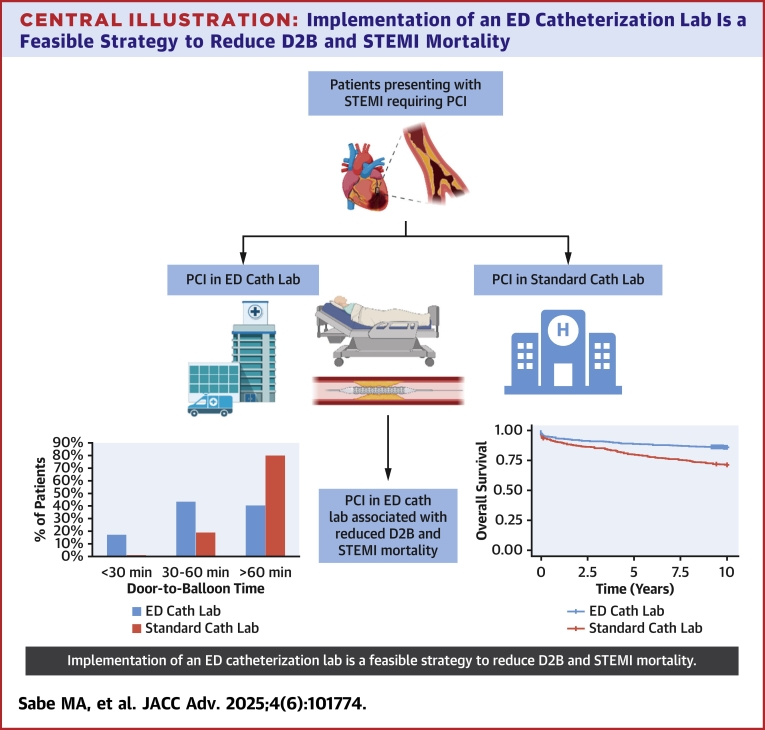
Implementation of an ED Catheterization Lab Is a Feasible Strategy to Reduce D2B and STEMI Mortality cath lab = catheterization lab; ED = emergency department; PCI = percutaneous coronary intervention; STEMI = ST-segment elevation myocardial infarction.

**Table 4:** Multivariable Cox Regression Model: Predictors of 10-Year Mortality

	HR	95% CI	*P* Value
ED cath lab	0.39	0.29-0.53	<0.0001
Age	1.04	1.03-1.06	<0.0001
Female	1.19	0.88-1.61	0.25
Race (White)	0.73	0.1-5.44	0.76
History of DM	1.81	1.35-2.44	<0.0001
History of CABG	1.1	0.7-1.72	0.68
IABP	1.1	0.79-1.51	0.59
Cardiogenic shock	2.63	1.75-3.94	<0.0001
LVEF <40%	2.22	1.66-2.97	<0.0001
3-4+ MR	1.20	0.83-1.73	0.33
HTN	1.05	0.8-1.39	0.71

CABG = coronary artery bypass grafting; cath lab = catheterization laboratory; DM = diabetes mellitus; ED = emergency department; HTN = hypertension; IABP = intra-aortic balloon pump; LVEF = left ventricular ejection fraction; MR = mitral regurgitation.

**Figure 4:**
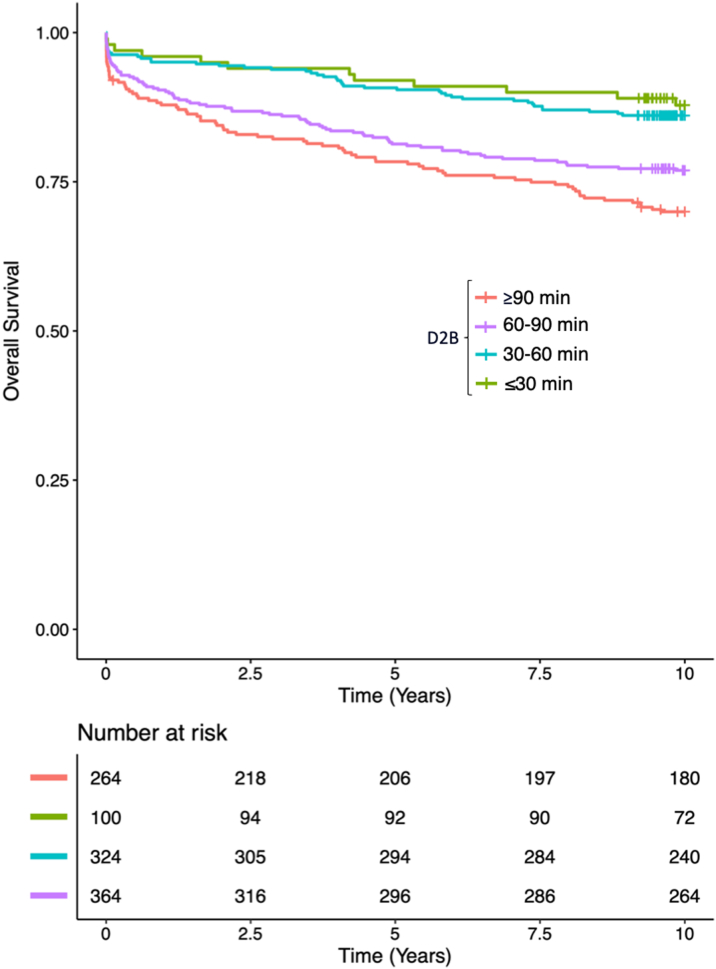
Cox Regression Demonstrating Categorical Door-to-Balloon Time and 10-Year Survival Cox proportional hazards regression plot shown for 10-year mortality of patients presenting with ST-segment elevation myocardial infarction undergoing percutaneous intervention categorized based on door-to-balloon time (D2B).

Lower D2B was found to be associated with improved survival (Figure 4). After adjusting for relevant covariates, D2B < 30 min and 30-60 min was associated with significant improvement in mortality at 10 years compared to D2B > 90 min (Table 5).

**Table 5:** Multivariable Cox Regression Model: Door-to-Balloon Time and 10-Year Mortality

	HR	95% CI	*P* Value
D2B (ref: >90 min)			
<30 min	0.35	0.19-0.67	0.001
30-60 min	0.48	0.33-0.70	0.0001
Age	1.04	1.03-1.05	<0.0001
Female	1.12	0.83-1.50	0.46
Race (White)	1.29	0.17-9.5	0.80
History of DM	1.83	1.36-2.47	<0.0001
History of CABG	1.0	0.64-1.58	0.97
IABP	1.21	0.88-1.66	0.25
Cardiogenic shock	2.43	1.62-3.66	<0.0001
LVEF <40%	2.04	1.53-2.72	<0.0001
3-4+ MR	1.31	0.92-1.89	0.14
HTN	0.96	0.72-1.27	0.76

CABG = coronary artery bypass grafting; D2B = door to balloon time; DM = diabetes mellitus; HTN = hypertension; IABP = intra-aortic balloon pump; LVEF = left ventricular ejection fraction; MR = mitral regurgitation.

## Discussion

In the present study, we sought to examine whether implementation of a catheterization laboratory in the emergency department for primary PCI improved door-to-balloon times and survival in patients presenting with STEMI to a community hospital. To our knowledge, this is the first reported study describing a cath lab in the ED, and we present the lowest published D2B time of 6 minutes. We found that patients treated in the ED cath lab had a significantly lower D2B time than patients treated in the standard cath lab, with 17% of patients having a D2B <30 minutes and a minimum D2B of 6 minutes. Of note, this cath lab did have a D2B as low as 5 minutes, which occurred after the analysis presented here.

D2B time of <90 minutes has been shown to be associated with decreased mortality in patients presenting with STEMI.^9^, ^10^, ^11^ Therefore, the American College of Cardiology/American Heart Association recommends a D2B of 90 minutes or less in these patients.^12^ However, there are conflicting data regarding D2B time. Menees et al^13^ found no significant difference in in-hospital and 30-day mortality in a large national registry (CathPCI Registry) despite improvements in D2B time, and they suggest that improved outcomes may be a reflection of institutional factors and/or operator experience. That particular study did not assess longer-term survival, and the authors note that this is a limitation of the study and that benefit may be seen in the long term.^13^ Our study demonstrated improved 30-day mortality, 1-year mortality, as well as long-term mortality up to 10 years. After adjusting for relevant covariates, our study also found that older age, history of DM, LVEF <40%, and cardiogenic shock on presentation were significant independent predictors of mortality in this patient population.

The long-term benefit of shorter D2B time may be related to improved long-term left ventricular function, decrease in scar formation, and possibly less scar-mediated sudden cardiac death; however, these hypotheses would need to be assessed in future studies. The phrase “time is muscle” has been emphasized by experts as data have shown that early PCI can improve LV function.^14^

There are some practical issues to consider when building a cath lab in the ED. Our team had to obtain institutional as well as state support to allow for a smaller than average sized cath lab to be built within the ED only for use in emergent cases. In addition to size limitations, it is important to consider cost, maintenance, and education of ED staff. We did have ED nurses who were cross-trained for the cath lab, which allowed for immediate triage, evaluation, and preparation in the ED cath lab upon arrival. Although the ED cath lab and standard cath lab are no farther than 5 minutes apart, the majority of time saved stems from streamlined logistics of evaluation and transfer especially during off-hours when the cath lab team is not in-house. Although a 6-minute door-to-balloon time is not the norm, when a patient is able to be evaluated, triaged, and prepped upon arrival with a cath lab team already called en route, very short D2B times were able to be obtained. The cath lab team is on call from home and do not stay in-hospital for call. However, when pre-hospital ECG STEMIs are called by emergency medical technicians, there are instances when D2B can be quite short and under 10 minutes. There are other practical benefits in having an ED cath lab, for instance, with unstable patients, especially during off-hours, there are readily available ED physicians ready to intubate and further support patients from a critical care perspective. In addition, the ED cath lab at our institution was used for emergent transvenous pacers when necessary.

### Study limitations

This study had several limitations, including that it was an observational, single-center analysis. As opposed to tertiary care centers where a significant number of patients with STEMI are triaged in the prehospital phase (ie, from a referral hospital), most of the patients presenting at this community hospital, similar to other community hospitals, were self-presenters, which could account for the higher overall D2B times. However, this study emphasizes that an emergency chest pain center is a model that could enhance the care of patients presenting with STEMI at local hospitals. Another limitation is that we did not have data available about post-PCI LV function or heart failure, which could account for long-term outcomes. As opposed to death data, which were available from the social security death index (SSDI), these data were not readily available as not all patients continued to follow up at this hospital in the long term. Another limitation of this study is that patients were not randomized to the 2 different cath lab locations; however, there were similar numbers of patients in each group and very few differences in baseline characteristics. There is nonetheless risk of selection bias and residual confounding particularly with regard to allocation to ED vs standard cath labs. Although patients who presented to the standard cath lab were more likely to have heart failure with reduced ejection fraction, and thus may represent a sicker patient population and may have reflected a selection bias in referral, after adjusting for cardiogenic shock on admission, LM disease, and LVEF ≤40%, the use of the ED cath lab was independently associated with lower mortality. There were periods of time when the ED cath lab was closed for renovation, during which all patients were referred to the standard cath lab; however, this was a short time period overall and did not correlate specifically with the beginning or end of the study time period. The authors recognize that temporal changes in STEMI treatment protocols and technology can also impact D2B and mortality outcomes and look forward to evaluating the impact of the ED cath lab in contemporary cohorts. Finally, for the long-term follow-up, death data were assessed using medical record review and SSDI, which may have limitations if a patient was not recorded as expired in the medical record or SSDI.

## Conclusions

In this observational study, we found that implementation of a cath lab for primary PCI in the ED significantly improved D2B time as well as 30-day, 1-year, and long-term mortality in patients presenting with STEMI at a community hospital. To our knowledge, this is the first reported study to present this novel technique to reduce D2B times and subsequently improve survival in patients presenting with STEMI. Implementation of a cath lab in the ED is a feasible option for institutions with primary PCI and requires collaboration between interventional cardiology and emergency medicine teams.

Perspectives **COMPETENCY IN SYSTEMS-BASED PRACTICE:** Implementation of hospital-based strategies to reduce door-to-balloon time in the treatment of patients presenting with acute ST-segment elevation myocardial infarction is important to reduce cardiovascular mortality. We implemented a catheterization laboratory within the ED at our institution as a novel strategy to further reduce D2B time and improve access to immediate revascularization. We found that D2B was shorter in ED vs standard catheterization lab patients, and ED catheterization lab patients had lower short- and long-term mortality than standard catheterization lab patients.

**TRANSLATIONAL OUTLOOK:** These findings indicate that implementation of an ED catheterization lab is a feasible strategy which community hospitals can utilize to reduce D2B and STEMI mortality.

## Funding support and author disclosures

The authors have reported that they have no relationships relevant to the contents of this paper to disclose.
